# Understanding carboxysomes to enhance carbon fixation in crops

**DOI:** 10.1042/BST20253072

**Published:** 2025-06-25

**Authors:** Nghiem Dinh Nguyen, Loraine M. Rourke, Alexandra Cleaver, Joseph Brock, Benedict M. Long, Dean G. Price

**Affiliations:** 1 Biochemical Science and Biochemistry Division, Research School of Biology, 134 Linnaeus Way, Australian National University, Acton, ACT 2601, Australia; 2 Plant Science Division, Research School of Biology, 134 Linnaeus Way, Australian National University, Acton, ACT 2601, Australia; 3 ARC Centre of Excellence in Synthetic Biology, Sydney, NSW, Australia; 4 Discipline of Biological Sciences, School of Environmental and Life Sciences, University Drive, The University of Newcastle, Callaghan, NSW 2308, Australia

**Keywords:** carbon fixation, cyanobacteria, photosynthesis, Rubisco, synthetic biology

## Abstract

Carboxysomes are bacterial microcompartments that enhance photosynthetic CO_2_ fixation by encapsulating ribulose-1,5-bisphosphate carboxylase/oxygenase (Rubisco) within a high-CO_2_ environment. Their modular, self-assembling nature makes them attractive for synthetic biology applications, particularly their transplantation alongside functional bicarbonate (HCO_3_^-^) transporters into plant chloroplasts to achieve improved photosynthetic efficiency. Recent advances have deepened our understanding of carboxysome biogenesis, Rubisco organisation and shell function. However, key questions remain, including the precise shell mechanistic action, which is critical for functional integration into new hosts. Addressing these questions, as well as identifying suitable bicarbonate transporters and fine-tuning expression levels, will be essential to utilising carboxysomes and the cyanobacterial CO_2_-concentrating mechanism for enhanced photosynthetic efficiency in crops.

## Introduction

The carboxysome is a bacterial microcompartment (BMC), essential for carbon fixation in cyanobacteria and some chemoautotrophic bacteria [1–4]. These microcompartments encapsulate ribulose-1,5-bisphosphate carboxylase/oxygenase (Rubisco), the enzyme catalysing the first step in the Calvin–Benson cycle [5], and carbonic anhydrase (CA; [6–8]), which interconverts carbon dioxide and bicarbonate (CO_2_ + H_2_O ↔ HCO_3_^-^ +H^+^). Encapsulation of the CA results in a localised, high-CO_2_ environment surrounding Rubisco leading to near-saturating substrate supply [9,10]. The evolution of carboxysomal Rubiscos in high CO_2_ environments has resulted in enzymes with low specificity for CO_2_, but with higher catalytic turnover rates than their unencapsulated Form I counterparts [11]. This high catalytic turnover has driven recent work towards the goal of introducing the cyanobacterial CO_2_-concentrating mechanism (CCM), into plant chloroplasts, with carboxysomes as a central component, in order to achieve more efficient carbon fixation in crops [12–18].

**Figure 1:**
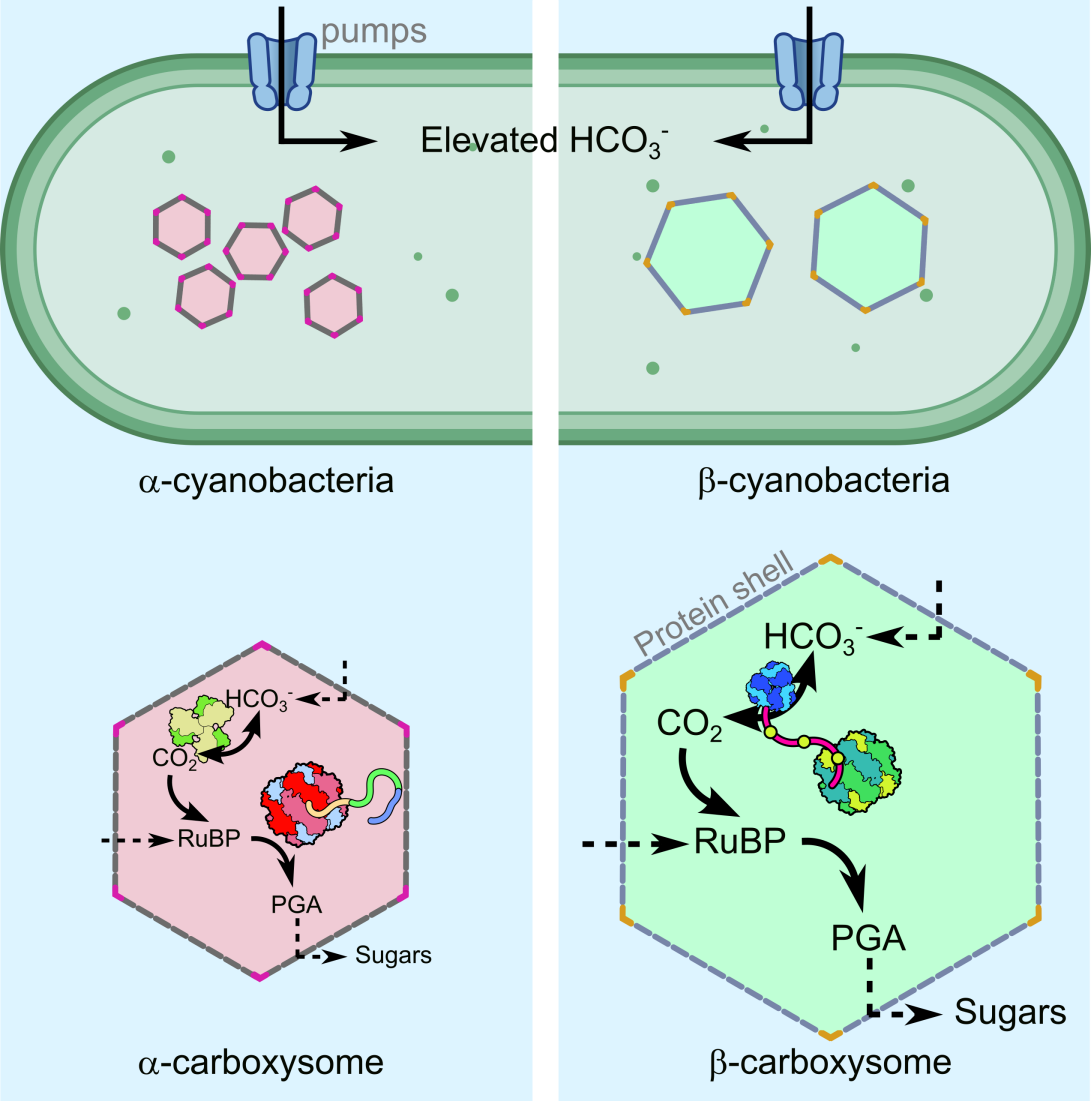
Schematic representation of cyanobacterial CO_2_-concentrating mechanism (CCM) in both α- and β-cyanobacteria. (**A)** Membrane-bound bicarbonate (HCO_3_^-^) transporters (coloured in light blue) facilitate accumulation of HCO_3_^-^ within the cyanobacterial cytosol. This accumulated HCO_3_^-^ subsequently diffuses into specialised microcompartments known as carboxysomes. In α-cyanobacteria, α-carboxysomes (pink) are typically smaller and more numerous than those found in β-cyanobacteria (green) [23]. (**B)** Despite structural differences between α- and β-carboxysomes, both serve similar functional roles. Across both carboxysome types, HCO_3_^-^ and ribulose-1,5-bisphosphate (RuBP) must pass through the selectively permeable carboxysome protein shell. Within the carboxysome, the carbonic anhydrase (CA) enzyme (yellow/green CsoSCA in α-carboxysomes; and blue CcaA/CcmM in β-carboxysomes) interconverts HCO_3_^-^ and CO_2_, raising the internal CO_2_ concentration for rapid fixation by ribulose-1,5-bisphosphate carboxylase/oxygenase (Rubisco). Here, the α-carboxysome Rubisco is shown in red, pink and blue. The β-carboxysome Rubisco is shown in green and yellow. The resulting product, 3-phosophoglycerate (PGA) exits the carboxysome for conversion into sugars within the Calvin cycle [5].

Carboxysomes are central to the cyanobacterial CCM, a system that likely evolved in response to the limited CO_2_ availability in a changing global atmosphere [10,19,20]. The cyanobacterial CCM consists of two key components; the carboxysome and membrane transporters that facilitate the movement of CO_2_ and bicarbonate (HCO_3_^-^) into the cell [21,22] (Figure 1). These transporter systems accumulate inorganic carbon (C_i_) as the relatively membrane-impermeable ion, HCO_3_^-^, at concentrations far exceeding the extracellular environment [24,25]. Bicarbonate is, however, able to diffuse through the selectively permeable carboxysome shell. Within the carboxysome, by action of the CA enzyme, a high-CO_2_ environment is formed, enabling rapid carbon fixation by Rubisco [10].

Early studies of carboxysome protein composition on isolated microcompartments from cyanobacteria [26] identified the key protein constituents, including Rubisco and CA [27,28]. Genetic approaches, including mutant screening and gene knockouts, were employed to identify the functional roles of these proteins, with key carboxysome protein mutants unable to grow at ambient CO_2_ concentrations [29–31]. Advances in synthetic biology technologies have since shifted research beyond identifying the composition of carboxysomes to understanding how proteins interact to enable regular and ordered carboxysome assembly. For example, recent structural studies have provided key insights into both carboxysome biogenesis [32–35] and shell architecture [36–40], deepening our knowledge of how these microcompartments assemble and how they regulate metabolite exchange.

The modular, self-assembling nature of α-carboxysomes makes them attractive platforms for designing custom reactors tailored to specific industrial and environmental applications [41]. However, a detailed understanding of both the structural and functional properties of carboxysomes is essential for their engineering into foreign hosts. This review highlights key findings and explores potential applications of carboxysomes in novel environments, particularly in addressing challenges related to enhancing the photosynthetic efficiency of C_3_ plants.

### Recent advances in understanding Rubisco structure and function

Rubisco is considered one of the most abundant and important proteins in plants, accounting for approximately 30–50% of total leaf protein [42]. This enzyme, widely found in chemoautotrophs and photosynthetic organisms including plants, cyanobacteria and algae, adopts several different structural configurations. The most common (Form I) Rubisco structure consists of eight large subunits capped by eight small subunits [5,43]. Despite its critical role in photosynthesis, Rubisco is commonly thought of as inherently inefficient as it poorly discriminates between CO_2_ and O_2_, thereby limiting its catalytic carboxylation efficiency [44]. To compensate for this limitation, plants produce Rubisco in large quantities, enabling net increased rates of catalysis through high concentration of active sites. Cyanobacteria, on the other hand, have evolved a CCM that encloses Rubisco within carboxysomes and elevates substrate CO_2_ concentrations at the active site as a means to maximise carboxylation.

Extensive kinetic catalogues have been constructed to explore the variation in not only Rubisco catalytic turnover rate but also enzyme CO_2_/O_2_ specificity. While early studies identified a loose correlation between these two traits [45,46], recent research has highlighted that phylogenetic constraints may play an important role in shaping Rubisco enzyme kinetic properties [9]. In addition to these evolutionary factors, machine-learning-based enzyme characterisation has provided additional insights [47]. While Rubiscos across all domains of life generally have low catalytic turnover rates, those encapsulated within carboxysomes tend to have higher catalytic turnover rates than their non-encapsulated counterparts [11]. However, they also have poorer CO_2_/O_2_ specificity and lower affinity for CO_2_ [11] meaning that such Rubiscos require the operation of a CCM to reach high productivity. By surveying the full diversity of Rubiscos, researchers can gain deeper insight into the characteristics of carboxysome-encapsulated Rubiscos and identify other Rubisco variants that may also be suitable for encapsulation [9,48].

In addition to understanding Rubisco kinetic traits, significant progress has been made with respect to understanding the assembly of the Rubisco holoenzyme structure. In most photosynthetic systems, considerable cellular effort is spent in ensuring correct folding and arrangement of Rubisco subunits to generate functional holoenzymes [49]. Across plants, cyanobacteria and green algae, a core of Rubisco large subunits is initially assembled before binding of the Rubisco small subunits to give the holoenzyme structure of eight large subunits and eight small subunits. Specific chaperonins prevent misfolding of the individual Rubisco large subunits, while Rubisco chaperones work to stabilise the core of eight Rubisco large subunits together [49,50]. Recent studies have solved the structure for various Rubisco chaperones, providing key insights into their mechanistic actions and the interacting interfaces which facilitate chaperone-assisted folding [51–54]. This understanding of Rubisco chaperone requirements has broader implications beyond carboxysome construction in foreign hosts. For instance, Whitney et al. [55] [54] demonstrated that chaperone inclusion for foreign Rubiscos significantly improved total Rubisco folding. Similarly, assembling plant Rubisco in *E. coli* required the co-expression of seven Rubisco chaperonins and chaperones, highlighting the complexity of this process and the engineering scale required for successful Rubisco assembly [56].

While structural and functional studies have separately provided valuable insights into different aspects of Rubisco, there is still a disconnect in understanding how Rubisco structure directly influences its functionality [47,57–59]. Recently, researchers have closely examined the biochemical properties of the Form II (a holoenzyme of two large subunits) *Rhodospirillum rubrum* Rubisco [48]. Here, they explored how specific residues influence key enzymatic properties such as catalytic turnover rate and CO_2_ affinity in a high throughput, wide sequence analysis in *E. coli* [48]. This integrated approach, coupled with the work of de Pins et al., [11] [46] is essential to understanding how Rubisco’s structure may influence its functionality, providing a platform for future efforts to re-engineer the enzyme for enhanced performance.

Complementary to structure–function studies, recent efforts have applied directed evolution approaches to engineer Rubisco variants with enhanced kinetic properties [60]. Using Rubisco-dependent growth assays in *E. coli*, researchers selected for enzyme variants with improved performance under defined conditions. One such strategy applied to a Form II Rubisco from *Rhodobacter sphaeroides* yielded mutants with up to a 27% increase in carboxylation rate and improved overall catalytic efficiency, while maintaining CO₂/O₂ specificity [61]. More recently, similar methods were extended to a red algal Rubisco, where modifications to active site residues led to significantly enhanced catalytic turnover *in vivo* [62]. These studies underscore how directed evolution can navigate natural trade-offs between activity, specificity and structural stability, while also revealing new sequence space for Rubisco improvement.

### Carboxysome assembly: from Rubisco condensates to functional microcompartments

Unlike plant Rubiscos that exist as free holoenzymes within the chloroplast stroma, cyanobacterial Rubiscos are encapsulated within carboxysomes. The high concentration of CO_2_ developed within carboxysomes has likely facilitated the evolution of Rubiscos with higher catalytic turnover rates [9]. Even so, two disparate carboxysome lineages (α-type and β-type; [3,19]) have been identified and are distinguished by the phylogenetic Form of Rubisco encapsulated within. Specifically, α-carboxysomes contain Form IA Rubisco, also found in chemoautotrophs, while β-cyanobacteria contain a lineage of the Form IB enzyme found in plants [19,63].

**Figure 2:**
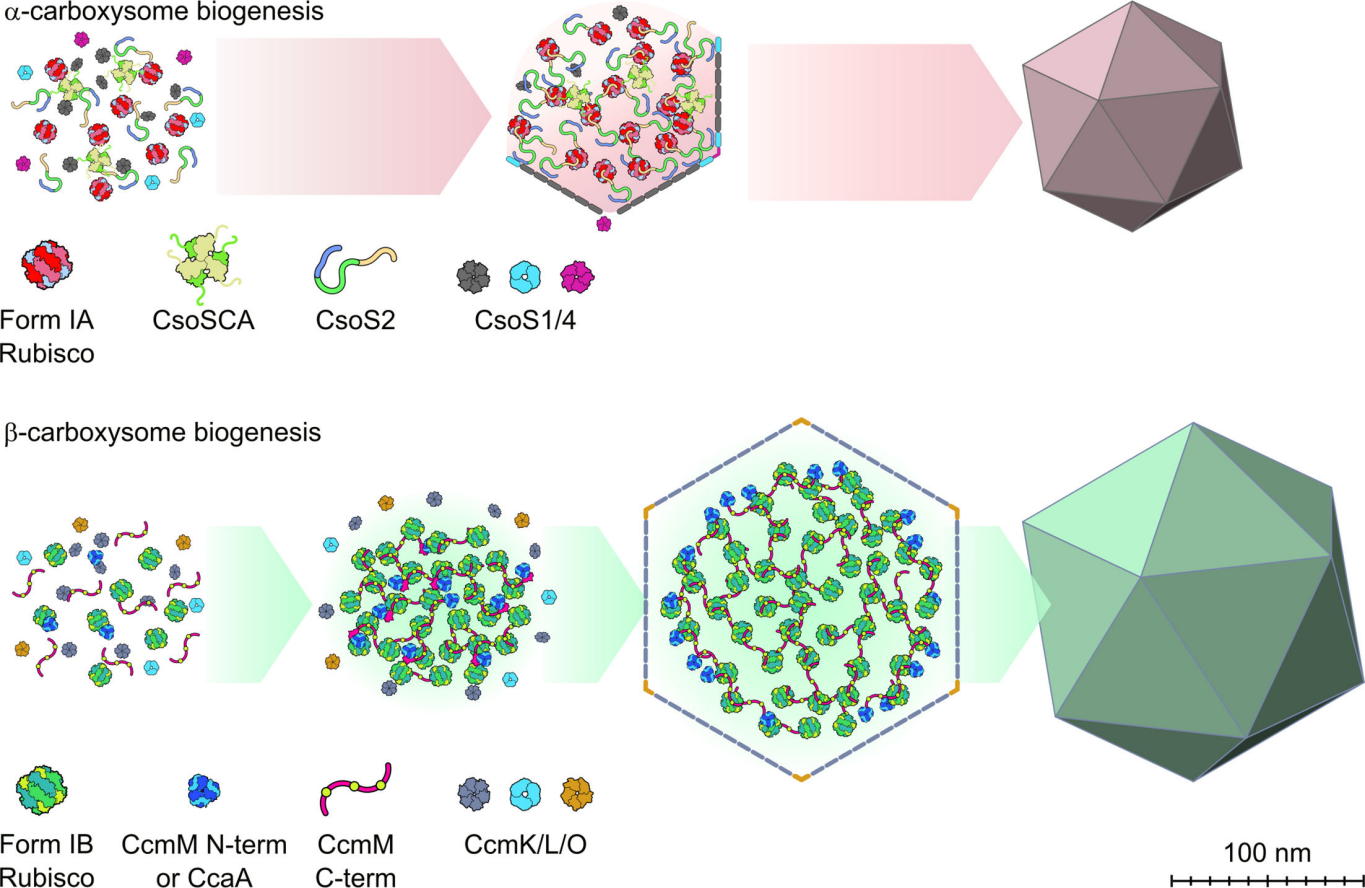
Schematic of the α- and β-carboxysome biogenesis pathways. Both α- and β-carboxysomes encapsulate a shared core of proteins within their lumen: ribulose-1,5-bisphosphate carboxylase/oxygenase (Rubisco), carbonic anhydrase (CA), the Rubisco-binding partner (CsoS2 in α-carboxysomes and CcmM in β-carboxysomes). Form IA Rubisco (found in α-carboxysomes; top) is shown in red and pink; Form IB Rubisco (found in β-carboxysomes; bottom) in green and yellow. The α-carboxysomal CA is depicted as a green and yellow hexameric unit, while the β-carboxysomal CA is shown as a blue hexameric unit. The α-carboxysome Rubisco-binding partner (CsoS2) is represented as a continuous ribbon of yellow, green, and blue; the β-carboxysomal binding partner (CcmM) appears as a red line with yellow globular domains. (**A**) α-carboxysome biogenesis involves the concomitant assembly of Rubisco condensates (formed between Rubisco, CsoS2 and CA, alongside shell assembly (hexameric shell proteins are coloured grey, while trimeric shell proteins are coloured blue and pentameric shell proteins are coloured pink) to produce intact carboxysomes. (**B**) For β-carboxysomes, this condensate formation appears to be a key step prior to shell protein recruitment (a so-called ‘inside-out’ formation pathway, hexameric shell proteins are coloured grey, while trimeric shell proteins are coloured blue and pentameric shell proteins are coloured pink).

Similar to the Rubisco biogenesis pathway wherein chaperonins and chaperones ensure the proper folding of Rubisco, there are several key steps required for successful Rubisco encapsulation within carboxysomes. Broadly speaking, Rubisco initially interacts with a cognate binding partner [34,35,64] and CA [6] to form Rubisco condensates, which subsequently interact with shell proteins to form intact, functional carboxysomes (Figure 2). In β-carboxysomes, this process tends to follow a more ordered, ‘inside-out’ pathway, with shell proteins encapsulating a preformed Rubisco condensate [32,33], whereas in α-carboxysomes, cargo and shell components assemble more concomitantly to produce intact and functional carboxysomes [65]. Significant progress has been made in elucidating the key protein interactions that drive this process, allowing researchers to re-engineer the carboxysome in *E. coli* and higher plants [66–68].

### Building blocks for carboxysome assembly

#### The β-carboxysome Rubisco binding partner, CcmM

The first step towards β-carboxysome assembly is the interaction between Rubisco and its cognate binding partner protein. In the β-carboxysome system, this is CcmM, a protein with two functional domains; a γ-CA-like domain at the N-terminus of the protein [7] and C-terminal repeating domains that bear homology to the Rubisco small subunit [69]. Owing to the presence of an internal ribosomal entry site within the *ccmM* gene transcript [70], two CcmM proteins can be expressed, a complete form (CcmM-long, or CcmM58 in *Synechococcus elongatus* PCC7942) that has both CcmM functional domains and a short form that only contains the repeating domains. Studies have shown that while the short form of CcmM (CcmM-short, or CcmM35 in *S. elongatus* PCC7942) primarily mediates Rubisco binding [35,71–73]. The presence of both CcmM forms in the correct stoichiometry is required to achieve regular carboxysome assembly [70].

The interaction between Rubisco and CcmM is an example of phase separation, a phenomenon where proteins demix from solution to form liquid droplets [35]. These droplets, commonly termed Rubisco condensates [74], are highly dynamic in nature and can be modulated by different environmental conditions [35]. For example, under reducing conditions, Rubisco condensates formed from Rubisco and CcmM-short were less dynamic than condensates under oxidising conditions [35]. Interestingly, CcmM-long can also drive condensate formation, though the composition of these condensates varies depending on environmental factors such as the redox state and the prevailing salt concentration [64]. While the biological significance of these condensates remains unclear, modelling has suggested that Rubisco condensates may have been an intermediate evolutionary step prior to carboxysome formation [10], and they also form the functional unit of Rubisco compartments known as pyrenoids found in many algal and some bryophytes [75,76].

#### The α-carboxysome Rubisco binding partner, CsoS2

In the α-carboxysome system, the Rubisco binding partner CsoS2 is an intrinsically disordered protein containing recognisable repeat domains and plays multiple roles in carboxysome assembly. Notably, the N-terminal region of CsoS2 mediates interactions with Rubisco, facilitating phase separation in a similar manner to CcmM in the β-carboxysome system [34]. The CsoS2 C-terminal domain interacts directly with the α-carboxysome shell, presumably playing a role in carboxysome structural integrity [77]. Additionally, the central repeating domains of CsoS2 help regulate carboxysome size and further reinforce shell stability [78,79].

Similar to CcmM in the β-carboxysome, the *csoS2* gene also encodes two proteins through a programmed ribosomal frameshifting event which sometimes leads to an early stop codon and apparent truncated N-terminal sequence or can be read through to generate the full-length protein [80]. Notably, this ‘slippery sequence’ is present in most proteobacterial *csoS2* sequences but absent from many α-cyanobacterial *csoS2* sequences [80]. Recent internal characterisation of α-carboxysomes using cryo-electron tomography has elucidated possible structural roles for both long and short form CsoS2 proteins. In carboxysomes of the α-cyanobacterium *Cyanobium*, which contains only the long form of CsoS2 [68,80], the protein was observed to interlink and organise Rubisco throughout the carboxysome [81]. On the other hand, in *Halothiobacillus neapolitanus* carboxysomes, where both the long (CsoS2B) and short (CsoS2A) forms of CsoS2 are present [80,82,83], the long form of CsoS2 protein was predominately associated with the carboxysome shell, while the truncated CsoS2 protein interlinked and organised Rubisco [81,84]. While both CsoS2 isoforms contribute to internal carboxysome organisation and shell structure, it remains unclear why some carboxysomes only require one CsoS2 form.

#### Comparmentalisation and regulation of carboxysomal carbonic anhydrase

Alongside Rubisco, CA plays an essential role within the carboxysome by interconverting HCO_3_^-^ and CO_2_ at a controlled rate [10]. To establish and maintain a high CO_2_ concentration within the carboxysome, the CA enzyme and its activity must be entirely confined within the carboxysome space. If the enzyme were to remain active outside the carboxysome in the cytosol, it would prematurely convert HCO_3_^-^ into CO_2_, leading to rapid dissipation of any accumulated HCO_3_^-^ and limited supply of C_i_ to the carboxysome [6,85,86]. A clear understanding of carboxysomal CA localisation (associated with the carboxysome shell or the lumen) remains to be elucidated. In *H. neapolitanus* α-carboxysomes, CA activity remains associated with the shell fraction when carboxysome Rubisco is mechanically released [87]. In β-carboxysomes, structural models suggest CA localisation at the shell [88], while functional models suggest an even distribution of CA within the carboxysome interior would be optimal for function [89]. The latter is consistent with evidence of a broad distribution throughout the carboxysome lumen [90]. The true location of carboxysomal CA is still to be unequivocally confirmed in both carboxysome types.

In α-carboxysomes, CA has been shown to interact directly with Rubisco via a motif that is also found in CsoS2 [6,91]. This has led to the hypothesis that CA may compete with CsoS2 for Rubisco binding [6,91]. Like CsoS2, the α-carboxysome CA has been observed to facilitate phase separation with Rubisco [6]. Interestingly, in photosynthetic α-cyanobacterial species, the CA has evolved a dependency on ribulose-1,5-bisphophsate (RuBP), hinting at additional regulatory mechanisms for the enzyme in fluctuating light environments, which typically lead to variable RuBP concentrations [8,92]. Structural studies have further elucidated the α-carboxysome CA architecture, with investigations of the *Cyanobium* PCC7001 CA revealing a trimer-of-dimer configuration [8]. Although earlier structural studies with a modified protein sequence revealed dimer formation in the canonical α-carboxysomal CA from *H. neapolitanus* [93].

In some β-cyanobacterial carboxysomes, the action of the CA is sometimes performed by the N-terminal γ-CA-like domain of the Rubisco binding partner, CcmM, with only a subset of species possessing a distinct β-carboxysome CA protein, CcaA [7,94,95]. Unlike their α-carboxysome counterparts, structural studies have shown that β-carboxysome CcaA can adopt both hexameric and tetrameric configurations [64,96]. Moreover, CcaA is modulated by environmental conditions, requiring Mg^2+^ for near maximum activity and becoming inactivated under reducing conditions [27]. Notably, β-carboxysome CcaA does not directly interact with Rubisco. Instead, it can be recruited into Rubisco condensates via the N-terminal domain of CcmM-long [64].

#### Deciphering the mechanistic action of the carboxysome shell

The carboxysome shell is a proteinaceous layer that surrounds the matrix of Rubisco and CA, establishing and maintaining a specialised microenvironment optimised for carbon fixation. In both α- and β-carboxysomes, the shell is predominantly composed of hexameric protein complexes with a central pore, while pentameric proteins occupy the vertices [1]. Minor shell proteins with larger pores have also been identified, likely facilitating alternative diffusion routes for specific metabolites [97–99]. Docked shell proteins have also been observed in β-carboxysome shells, which may also contribute to modulating metabolite transport via a gated pore mechanism [37,100]. Collectively, these architectural features highlight the permeability and structural robustness of the carboxysome shell, both of which are essential for carboxysome functionality.

**Figure 3:**
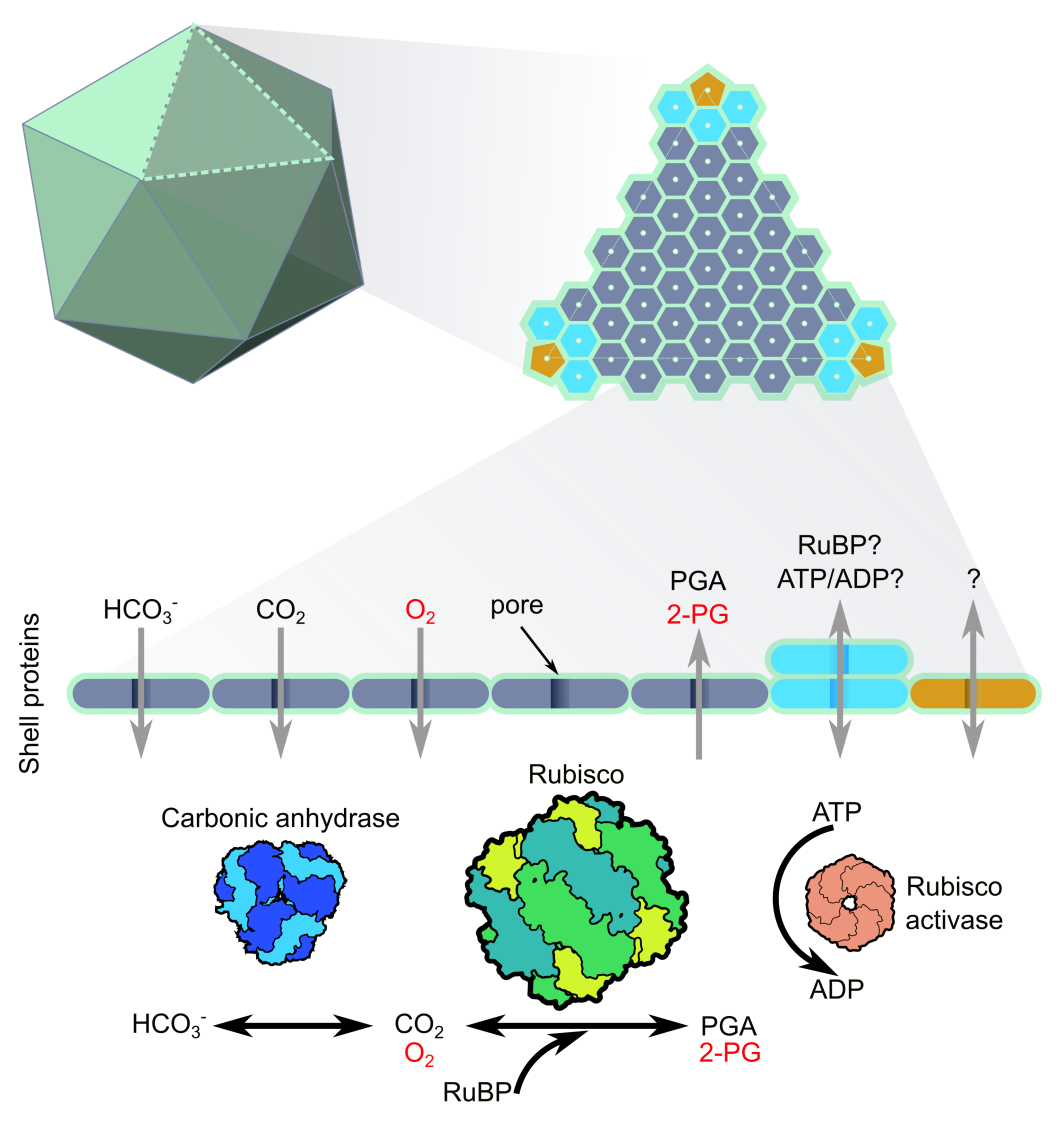
Schematic of the proposed mechanistic action of the carboxysome shell. The carboxysome face is composed of hexameric shell proteins (coloured dark grey and light blue) and pentameric shell proteins (coloured in brown), with pentamers occupying the vertices. The carboxysome shell protein encapsulates ribulose-1,5-bisphosphate carboxylase/oxygenase (Rubisco), carbonic anhydrase (CA) and Rubisco activase. Central pores in the shell proteins are thought to facilitate the selective transport of small molecules, with the hexameric pores implicated in the movement of bicarbonate (HCO_3_^-^), CO_2_ and O_2_. Notably, gaps between shell proteins have the capacity to allow small molecular passage [36,39,40,101]. Protons are expected to have easy passage across the shell [102,103] and play a significant role in modulating carboxysome function (not shown [10,104]). The mechanisms by which the Rubisco reaction products phosphoglycerate (PGA) and 2-phosphoglycolate (2 PG) traverse the carboxysome shell remains unknown. Additionally, key metabolites such as ribulose-1,5-bisphosphate (RuBP), adenosine triphosphate (ATP) and adenosine diphosphate (ADP) which are required by Rubisco and Rubisco activase, must cross the carboxysome shell but the transport routes for these substrates require further elucidation. To date, there is no evidence that there is selective discrimination of CO_2_ and O_2_ at the shell, while modelling suggests the elevation of CO_2_ within the carboxysome by CA action is sufficient to reduce Rubisco oxygenation [10]. Coupled shell proteins may act as gated pores, allowing shuttling of larger molecules in and out of the carboxysome [97,105,106].

As a selective barrier, the carboxysome shell probably regulates the movement of essential metabolites (Figure 3). The substrates HCO_3_^-^ and RuBP must enter the carboxysome, while the carboxylation product 3-phosphoglycerate (3-PGA) needs to exit efficiently to enter the Calvin cycle. Conversely, there is an expected limitation to CO_2_ passage across the shell to provide sufficient diffusional resistance to CO_2_ efflux that enables its accumulation. Early structural studies of the shell’s hexameric and pentameric protein complexes provided insights into how metabolites might traverse the shell, revealing central pores that could serve as pathways for metabolite transport [36,39,40,107].

Building on these findings, computational modelling has predicted the energetic favourability of metabolite movement across the shell. For instance, simulations have demonstrated bicarbonate permeation through the central pore of the primary hexameric shell protein is energetically favourable (Figure 3) [108]. More recently, molecular dynamics modelling of metabolite diffusion through a simplified β-carboxysome shell (consisting only of the major hexameric shell proteins and the pentameric vertex proteins) revealed that the shell is highly permeable to both CO_2_ and O_2_, with both preferentially diffusing between the interfaces of hexameric and pentameric shell proteins [101]. Notably, this simplified carboxysome shell also reflects CO_2_ back into the carboxysome lumen, thus sustaining the high internal CO_2_ concentration [101]. These findings are of particular significance, since they imply that no specific diffusional resistance is required to prevent O_2_ entry to the carboxysome, but that it is the accumulation of CO_2_ that limits the Rubisco oxygenation reaction [10].

Additionally, this molecular dynamics model [101] showed that bicarbonate and 3-PGA may primarily transit through the central pores of hexameric shell proteins, whereas RuBP showed minimal movement across this simplified shell (Figure 3) [101]. This suggests that a minor shell protein excluded from the simplified model could be the primary facilitator of RuBP transport. Notably, the kinetics of RuBP use by isolated α-carboxysomes suggests a diffusional resistance to its entry [68], but the mechanism of transit is yet to be identified. It is also relevant to note the potential requirement of adenosine triphosphate (ATP) within the carboxysome to fuel Rubisco activase function [109], which has been identified in native α-carboxysomes using mass spectrometry [110], highlighting that potential gated pores may play roles in moving larger metabolites in and out of the compartment. Further insights into metabolite passage across carboxysome shells are needed to clarify function and enable more accurate compartment modelling.

#### Critical gaps in carboxysome research

While significant progress has been made in elucidating the mechanisms underpinning Rubisco encapsulation and carboxysome shell architecture, key knowledge gaps remain. One example is the functional role of the β-carboxysome protein CcmN, with only a few copies of this protein observed in β-carboxysomes [111]. When the *ccmN* gene was knocked-out, cyanobacterial mutants were unable to grow at ambient CO_2_ levels, producing dense polar bodies rather than regularly structured carboxysomes and indicating an essential functional role [112]. Initial examination via yeast hybrid studies has found CcmN, which contains an C-terminal encapsulation peptide [112], interacts with both the β-carboxysome shell and CcmM-long [71]. Altogether, this strongly suggests that CcmN plays a role in mediating interactions with the β-carboxysome shell [112,113] despite a very low abundance in the carboxysome [114].

Another unresolved area relates to the regulation of Rubisco activity within carboxysomes. While many photosynthetic organisms rely on Rubisco activases to remodel inhibited Rubisco complexes [49], protein homologues are inconsistently present across cyanobacteria [115], raising the possibility of alternative regulatory mechanisms or reduced susceptibility to inhibition in the carboxysomal context. Notably, where Rubisco activases [109] and other ATP-dependent processes operate within carboxysomes, they necessitate a reliable supply of ATP. However, how ATP and other key metabolites traverse the selectively permeable carboxysome shell remains unclear. Although recent molecular dynamics simulations and structural studies have begun to explore shell permeability [101], the precise mechanisms of metabolite exchange and their regulation are still not fully resolved.

### Re-engineering carboxysomes in heterologous expression systems

Reconstituting intact and functional carboxysomes in *E. coli* has been achieved [67]. The development of high-throughput molecular cloning strategies, such as DNA assembly techniques [116–118], has greatly facilitated the assembly of multigene expression cassettes necessary for producing proteins required for both Rubisco and carboxysome assembly. The use of such techniques is particularly important, given the requirement to control relative protein expression levels to successfully assemble carboxysomes. This was examined using a newly engineered *E. coli* cell line CCMB1, developed to grow in ambient CO_2_ levels when a functional CCM is present [67]. Initial attempts to express a plasmid containing all necessary *H. neapolitanus* carboxysome genes in the engineered CCMB1 *E. coli* line failed to produce carboxysomes [67]. However, after several rounds of evolution, mutations in the promoter and terminator sequences around these core carboxysome genes led to the successful assembly of carboxysomes in *E. coli* [67]. Insights gained from *E. coli* carboxysome assembly offer valuable lessons for future attempts to achieve carboxysome assembly *in planta* [17]. In recent work, where most of the carboxysome genes were expressed in tobacco, re-engineered carboxysomes were observed to exhibit a markedly different CsoS2 protein stoichiometric ratio compared to their native counterparts, highlighting the need for further optimisation in plant systems [66].

Recent advances in synthetic biology and our deepening understanding of carboxysome architecture have also paved the way for constructing hybrid carboxysomes that combine advantageous features from different carboxysome systems [119,120]. For example, the *Cyanobium* shell is composed of a single hexameric shell protein isoform (CsoS1A), whereas the shells of other α-cyanobacterial species can include up to three distinct isoforms (CsoS1A, B and C) [121]. Additionally, significant variation exists in the catalytic turnover rates of encapsulated Rubisco among cyanobacteria, with one of the fastest Rubiscos found in *Synechococcus* WH8102 [11,122]. By engineering a carboxysome with simple architecture that encapsulates this faster Rubisco, it is theoretically possible to engineer a hybrid carboxysome with improved performance [119,120,123]. However, while these engineered hybrids hold considerable promise, it is essential to verify their functionality closely mirrors that of a regular carboxysome, which can be determined using the newly engineered *E. coli* cell line CCMB1 [67].

### Potential for the use of carboxysomes to achieve biotechnology engineering goals

The faster catalytic rate of carboxysome-encapsulated Rubiscos has driven efforts to replicate the cyanobacterial CCM within C_3_ plants, with full integration projected to increase photosynthetic efficiency by up to 60% [14]. This CCM would theoretically outperform a CCM of a C_4_ plant [124]. However, successfully replicating this system presents several challenges which include ensuring proper Rubisco folding, encapsulation within an intact carboxysome [15] and coordinated HCO_3_^-^ accumulation through correctly localised and functional transporters [22]. Despite these complexities, significant progress has been made in this area, demonstrating the feasibility of this ambitious biotechnological goal [15].

Cyanobacterial Rubisco has been successfully expressed in *Nicotiana tabacum*, with co-expression of its cognate chaperones increasing soluble Rubisco yield [125,126]. Furthermore, when β-cyanobacterial Rubisco was co-expressed with its cognate binding partner, condensates were observed, suggesting an initial step toward microcompartment formation [126]. The introduction of major carboxysome shell components led to the assembly of simplified α-carboxysomes in tobacco, albeit with some irregular structures [68]. More recently, the inclusion of further shell proteins alongside CA has resulted in the formation of α-carboxysomes which are lacking minor shell components. This marks a key step towards integrating a functional cyanobacterial CCM into plants [66,121].

To date, efforts to introduce the carboxysome into *N. tabacum* have primarily relied on chloroplast transformation, as demonstrated by the successful replacement of Rubisco in a tobacco master line with carboxysome-associated genes [127]. However, this approach is not easily applicable to other plant species due to the limited availability of chloroplast transformation techniques. As such, some attempts have focused on delivering carboxysome proteins to the chloroplast from nuclear encoded carboxysome genes [128]. However, this comes with significant complexity, requiring efficient protein delivery with appropriate signal peptides for each carboxysome component. Signal peptide cleavage and uninterrupted, time-critical folding of proteins is crucial to ensure these mega-Dalton complexes are brought together in a functional manner. Consequently, establishing proof-of-concept through chloroplastic transformation in amenable species remains the current priority and has already proven to be somewhat achievable [66,68,126]. In parallel, alternative strategies including nuclear transformation could be explored for gene integration, understanding the complexities of this approach. Additionally, the emerging use of plastid mini-chromosomes presents a promising strategy for introducing carboxysome-related genes into diverse plant systems and may represent the most promising approach for future studies [129].

### Additional considerations to successfully implement the cyanobacterial CCM in plant chloroplasts

Importantly, it must be plainly stated that adding carboxysomes to the chloroplasts of C_3_ crop plants, without elevated chloroplastic HCO_3_^-^, is entirely incapable of improving photosynthetic function [68]. Thus, beyond carboxysome assembly, reconstituting the cyanobacterial CCM in plant chloroplasts requires additional modifications, the most critical of which is a mechanism to elevate chloroplastic HCO_3_^-^ [15]. One critical challenge that must be addressed is the presence of native CAs in the chloroplast stroma [130,131], which must be eliminated to prevent the premature conversion of HCO_3_^-^ into CO_2_ outside the carboxysome [86]. Recent work has successfully eliminated major CAs in the chloroplast stroma of both *N. tabacum* tobacco and *Arabidopsis thaliana* [132,133], and such mutants have been critical in developing methods to assess chloroplastic HCO_3_^-^ uptake function *in planta* [133–135].

Additionally, a localised and functional HCO_3_^-^ transporter must be introduced into the chloroplast inner envelope membrane (IEM) to ensure HCO_3_^-^ supply. As noted above, the absence of this critical component renders a chloroplastic carboxysome non-functional. However, integrating these transporters has been challenging, as their removal from their native context disrupts regulatory mechanisms required for functionality [136]. To bypass this, directed evolution approaches have been used to overcome the lack of functionality that is often observed in heterologously expressed HCO_3_^-^ transporters [135–137].

### Closing remarks

There remain several unknowns that, if answered, would be valuable for realising the full potential of carboxysomes expressed in foreign systems. Continued research into the regulatory mechanisms underlying cyanobacterial CCM will be essential for future efforts to re-engineer this system into heterologous hosts. Furthermore, as technological progress is made towards transplanting the cyanobacterial CCM into plants, identifying a suitable HCO_3_^-^ transporter and developing robust genetic integration techniques for diverse plant species will be crucial. Altogether, addressing these challenges will pave the way to improve terrestrial plant photosynthesis utilising the cyanobacterial CCM.

**Perspectives**

- The cyanobacterial CO₂-concentrating mechanism (CCM) elevates CO₂ concentrations around the Rubisco enzyme which is encapsulated within a microcompartment called the carboxysome. This enables the cyanobacterial Rubisco to exhibit a higher catalytic turnover rate relative to its plant counterparts.
- Recent research has elucidated key aspects of the carboxysome biogenesis pathway, namely Rubisco assembly and the key interaction between Rubisco and its cognate carboxysome binding partner. Advances in structural biology have also provided valuable insights into the mechanistic function of the carboxysome shell.
- As our understanding of the cyanobacterial CCM becomes increasingly comprehensive, the targeted introduction of the carboxysome and functional bicarbonate transporters into crop plants may offer a viable strategy for enhancing photosynthetic efficiency.

## Abbreviations

ADP: adenosine diphosphate
ATP: adenosine triphosphate
BMC: bacterial microcompartment
CA: carbonic anhydrase
CCM: CO₂-concentrating mechanism
IEM: inner envelope membrane
3-PGA: 3-phosphoglycerate
RuBP: ribulose-1,5-bisphophsate
Rubisco: ribulose-1,5- bisphosphate carboxylase/oxygenase
